# Applications of Yeast Synthetic Biology Geared towards the Production of Biopharmaceuticals

**DOI:** 10.3390/genes9070340

**Published:** 2018-07-06

**Authors:** Roy S. K. Walker, Isak S. Pretorius

**Affiliations:** 1 Department of Molecular Sciences, Macquarie University, Sydney 2109, Australia; 2 Chancellery, Macquarie University, Sydney 2109, Australia; sakkie.pretorius@mq.edu.au

**Keywords:** biopharmaceuticals, biologics, natural products, recombinant proteins, *Saccharomyces cerevisiae*, synthetic biology, yeast

## Abstract

Engineered yeast are an important production platform for the biosynthesis of high-value compounds with medical applications. Recent years have witnessed several new developments in this area, largely spurred by advances in the field of synthetic biology and the elucidation of natural metabolic pathways. This minireview presents an overview of synthetic biology applications for the heterologous biosynthesis of biopharmaceuticals in yeast and demonstrates the power and potential of yeast cell factories by highlighting several recent examples. In addition, an outline of emerging trends in this rapidly-developing area is discussed, hinting upon the potential state-of-the-art in the years ahead.

## 1. Yeast Synthetic Biology Shifts Biopharmaceutical Production Capabilities into High Gear

The natural world hosts a vast reserve of chemical complexity which humans have harnessed since ancient times for medical benefit. The first likely recorded use of medicine dates back tens of thousands of years with the discovery of pollen from medicinal flowers on Neanderthal bones [1]. More recently, humans have isolated the active compounds responsible in medicines, such as the discovery in 1804 of morphine as the active ingredient of opium [2] and the antibacterial properties of penicillin in 1928 by Alexander Fleming [3]. Today, biopharmaceutical compounds may be extracted from plants, although such methods are often environmentally destructive or uneconomical due to the inherently low titres found in nature [4]. As with non-biological pharmaceuticals, biopharmaceuticals may also be manufactured through chemical synthesis. However, chemical synthesis is often uneconomical due to the structural complexity of natural products and usually requires toxic solvents, harsh conditions and the generation of by-product wastes [5]. Indeed, chemical synthesis in the overall pharmaceutical industry generates more waste products per ton of product than oil refining, bulk chemicals and fine chemicals [6].

The ability to heterologously produce biopharmaceuticals in non-native biological host cells has several advantages over plant extraction and chemical synthesis. Aside from reduced environmental harm, advantages include increased scope for the biosynthesis of complex molecules [7], reliable production supply chains [8] and improved scalability for compounds that normally exist at low levels in nature [9]. In recent years, capabilities have significantly expanded with the development of modern DNA sequencing methods and bioinformatics analysis which have enabled elucidation of numerous new genes and pathways [10,11]. However, it is the even more recent advent of synthetic biology which has revolutionised capabilities in modern biotechnology. In contrast to traditional genetic engineering, synthetic biology has significantly greater capacity to design, construct and test biological systems with higher throughput and potential for automation.

Host organisms for the heterologous production of biopharmaceuticals include *Escherichia coli*, mammalian cells, yeasts and plants. Each host organism has inherent advantages and disadvantages (summarised in a review on recombinant protein production [12]). For example, *E. coli* displays rapid growth, but may form endotoxins and inclusion bodies, and is not capable of protein glycosylation. Mammalian cells are good for post-translational modification and display high yields from Chinese Hamster Ovary cell lines, but are hindered by a slow doubling time, require complex media and may be subjected to viral contamination. Plant cells display the advantage of photosynthesis in response to light, but again are slow growing and are less amenable to genetic modification.

**Figure 1:**
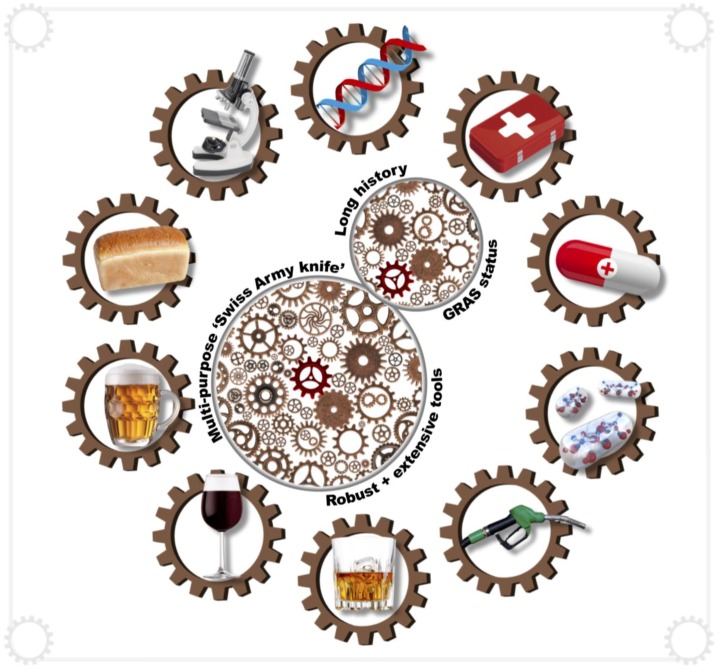
The budding yeast *Saccharomyces cerevisiae* is a multi-purpose single-celled fungus with a long history in the fermentation industry. Although several yeast species have distinct individual biotechnological applications, *S. cerevisiae* serves as the dominant eukaryote in industry and research. This unicellular eukaryotic research model and multi-purpose host organism, with generally regarded as safe (GRAS) status, has numerous applications. On the one hand, this *Swiss Army knife* yeast is a centralising cog that inter-connects fundamental eukaryotic research in numerous laboratories across the world; and, on the other hand, it is the fermentation industry’s fundamental cog driving the production of a broad range of fermented foods, beverages, biofuels and pharmaceutical products.

Yeast cells have several important advantages for biopharmaceutical production. Aside from having been used by humans for millennia for the production of bread and alcoholic beverages, yeasts are now used as cell factories for the production of biofuels, biocontrol agents, probiotics, flavouring agents and a host of high-value products [13] (Figure 1).

Yeast cells display many beneficial characteristics as cell factories, including robustness in the fermentor, host an extensive repertoire of genetic tools, require inexpensive growth media, and many species are generally regarded as safe (GRAS). Common industrially-used species include the ubiquitous *Saccharomyces cerevisiae*, as well as *Pichia pastoris* (Syn. *Komagataella phaffii* [14]), *Yarrowia lipolytica* and *Kluyveromyces marxianus*.

Applications of synthetic biology for the production of therapeutic proteins in a range of yeast hosts were recently reviewed [15], in addition to synthetic biology applications to optimise yield of therapeutic natural products and to identify new natural or unnatural therapeutic products [16]. This minireview briefly summarises the importance of identifying the optimal yeast host before presenting a concise overview of synthetic biology approaches to improve biopharmaceutical production in yeast. Recent developments in the application of yeast synthetic biology are then highlighted, before a discussion on emerging trends in this rapidly-developing area.

## 2. The Importance of Identifying the Optimal Cell Factory for Biopharmaceutical Production

**Figure 2:**
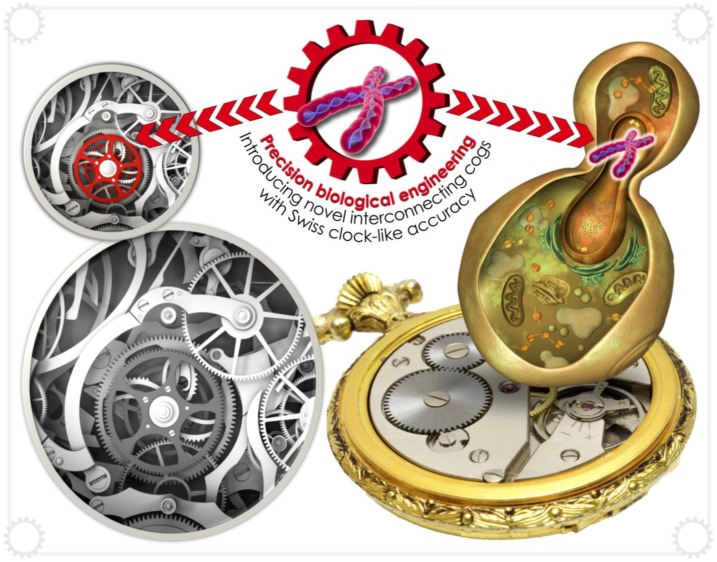
*Saccharomyces cerevisiae* is the predominant yeast species used in synthetic biology. Its well-characterised genome, well-understood physiology and powerful homologous recombination machinery has facilitated the development of numerous synthetic biology tools, enabling incorporation of heterologous metabolic pathways. The synthetic biologist may be described as a skilled watchmaker, applying the concerted action of these tools and pathways which interact with *Swiss clock*-like accuracy and synchronization.

*Saccharomyces cerevisiae* remains the predominant yeast species used in synthetic biology, primarily due to its well-characterised genome, well-understood physiology, powerful homologous recombination machinery and a well-developed set of synthetic biology tools [17] (Figure 2). However, other nonconventional yeast species display beneficial characteristics as cell factories, such as the ability to secrete large quantities of protein (*P. pastoris* and *Pichia angusta* (*Hansenula polymorpha*)) [18,19], grow rapidly and at high temperature (*K. marxianus*) [20] and produce high levels of lipids (*Y. lipolytica*) [21]. Indeed, *Y. lipolytica* has been engineered to produce saturated cells containing up to 90% lipid content [22]. The choice of chassis organism is therefore an important one to make and may have a significant bearing on not only final yield, but also the rate of biopharmaceutical production—an important requirement to limit the length of time before reaching optimum titre.

An important consideration for the biosynthesis of recombinant therapeutic proteins is glycosylation, a post-translational modification often required for proper protein folding and function. The glycosylation machinery in humans differs to that of yeast, with the latter displaying a tendency to hyper-mannosylate proteins [23]. These high-mannose, hyperglycosylated structures are also likely responsible for the antigenic properties of many proteins produced by yeast [23] and their short serum half-lives [24], rendering them unsuitable for use in human therapies. Several attempts have been undertaken to humanise the glycosylation machinery, especially that of *P. pastoris* due to its high protein yield, shorter glycosylation chain lengths [25] and the absence of immunogenic terminal α-1,3-linked mannose residues present in *S. cerevisiae* [26]. Humanisation of glycosylation in a range of yeast species generally first requires reducing hyperglycosylation by deleting the *OCH1* gene, which encodes α-1,6-mannosyltransferase responsible for triggering heterogeneous hypermannosylation [26] (which is discussed in more detail elsewhere [15]). Further alterations to glycosylation then require the introduction of several enzymes including glycotransferases and glycosidases (reviewed in further detail [23]).

A further important consideration for many non-*S. cerevisiae* yeast species is a preference for the non-homologous end joining pathway (NHEJ) in lieu of the highly-efficient homologous recombination machinery in *S. cerevisiae* for DNA repair [27]. The absence of efficient homologous recombination (HR) greatly limits the amenability for genetic manipulation and associated ability to incorporate exogenous synthetic DNA. However, some efforts have been undertaken to improve the efficiency of HR in these species. For instance, the *KU70* gene may be removed from *P. pastoris*, increasing the efficiency of HR by up to 100% [28]. This approach has also been applied to other yeast species, including *P. angusta* [29] and *Y. lipolytica* [30].

## 3. Synthetic Biology Tools for Heterologous Production of Biopharmaceuticals

**Figure 3:**
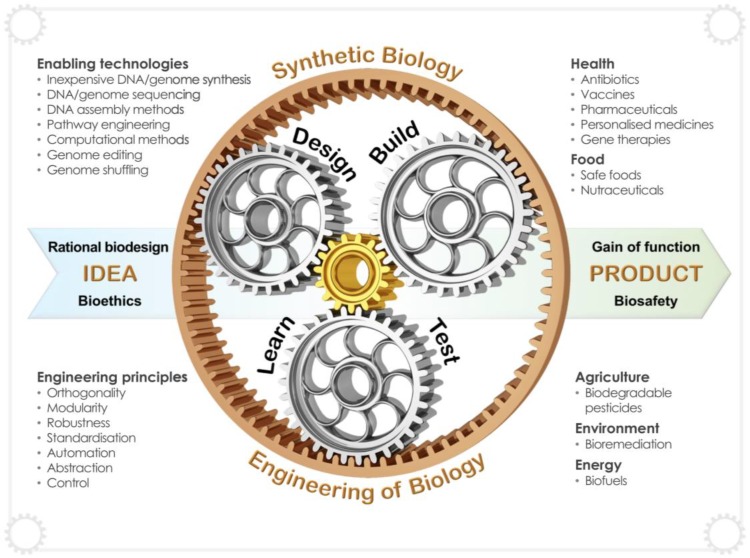
Synthetic biology entails the engineering of biology, incorporating enabling technologies and enabling approaches framed around rational engineering principles. As opposed to classical biological tinkering, synthetic biology can rapidly translate an idea into a product or application through the recursive Design-Build-Test-Learn cycle.

Synthetic biology is an enabling technology that aims to increase predictive capacity and reconcile complexity in biological systems. This field generally applies rational engineering principles, usually framed around the ubiquitous Design-Build-Test(-Learn) cycle, to rapidly develop gain-of-function in living systems. The Design-Build-Test cycle represents the primary three cogs which accelerate the translation of an idea into a product, application or cellular gain of function (Figure 3). Although several definitions exist, synthetic biology may be defined simply as *the engineering of biology*; incorporating principles and tools such as orthogonality, modularity, automation, standardisation, robustness and a strong emphasis on rational design [31]. Largely driven by technological advances in DNA synthesis, corresponding decreases in cost, advances in computational methods and the elucidation of complex metabolic pathways, synthetic biology has significantly advanced our ability to produce complex natural products in yeast. This section briefly summarises several synthetic biology approaches that may be used to improve biopharmaceutical production in yeast. This topic has previously been reviewed in greater depth [16].

Due to the inherent codon bias between different organisms, a common approach is to re-code genes (synonymous recoding) to improve translation efficiency. For instance, it was demonstrated that the recoding of the Igk chain protein from mouse (*Mus musculus*) for production in *S. cerevisiae* led to a 50-fold increase in protein yield [32]. It should be noted that other dimensions of codon bias do exist, and matching codon usage to general tRNA abundance may not necessarily impart the highest yield (reviewed in greater depth [33]). For example, it may be beneficial to optimise codon usage to genes expressed under the specific condition of interest, such as those expressed highly during stationary-phase growth, pH, carbon source or respiration amongst others [34]. Several iterations of the same gene may therefore be required to optimise yield.

A powerful approach used in synthetic biology is the application of modularity. The commonly-applied *Golden Gate* assembly method uses Type IIS restriction enzymes for modular one-pot DNA assembly [35], and may be used to rapidly construct and screen promoter-gene pairs to identify the optimal configuration for gene expression. *Golden Gate* assembly has previously been used to optimise production of benzylpenicillin in *S. cerevisiae* [36] and to optimize production of β-carotene in *Y. lipolytica* [37]. A disadvantage of such systems is the occurrence of internally-cutting restriction sites, which may necessitate removal through synonymous recoding.

Further enabling technologies include genome-editing tools, such as the powerful CRISPR/Cas9 [38], which may be multiplexed to introduce several nucleotide modifications simultaneously. CRISPR has also recently been applied to introduce a library of genetic parts into the yeast genome to rapidly develop gain-of-function in an automated manner [39]. Comparatively newer developments also include CRISPR/Cpf1 [40].

As opposed to optimising metabolic pathway function at the genetic level, a promising approach includes control of enzyme behaviour at the protein level. Compartmentalisation may be used to spatially and temporally separate key metabolic enzymes and locally optimise their behaviour. Localising pathway enzymes to intracellular compartments may be used to increase local substrate concentration, prevent efflux or diffusion of pathway intermediates, prevent interference from competing pathways, reduce intermediate toxicity or spatially separate unwanted side-reactions to allow spontaneous product formation to occur [11]. Synthetic, non-endogenous organelles were recently constructed in *S. cerevisiae* [41], which represent ideal microcompartments for enzyme function isolated from the host cell machinery. The nanocomparments, based on self-assembling encapsulin proteins from the bacterium *Myxococcus xanthus*, were shown to protect cargo proteins from protease degradation and act as nanoreactors for enzyme function.

Synthetic biology technologies are most mature in *S. cerevisiae*. However, in recent years we have witnessed the development of several new synthetic biology approaches in non-standard yeasts, such as promoter engineering in *P. pastoris* [42,43], an inducible promoter system in *Y. lipolytica* [44] and CRISPR/Cas9 in *P. pastoris* [45]. Deaminase-mediated targeted point mutagenesis (Target AID) was also recently used to improve the efficiency of homologous recombination in *K. marxianus*, facilitating subsequent introduction of the CRISPR/Cas9 system [46].

## 4. Recent Developments in the Production of Biopharmaceuticals

**Figure 4:**
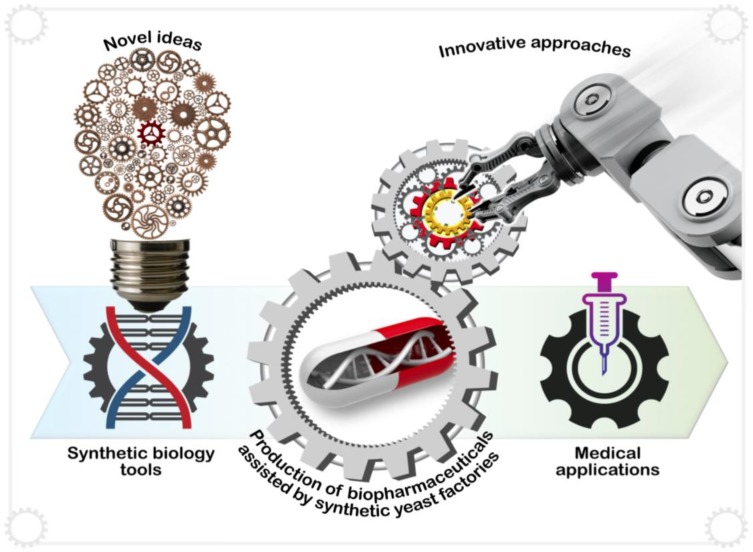
Imaginative ideas and innovative approaches are spurring advances in the application of synthetic biology to produce biopharmaceuticals in yeast. Several recent pioneering studies have demonstrated the capacity of synthetic yeast cell factories for the biosynthesis of complex natural products with promising medical applications. The reconstruction of a de novo noscapine biosynthetic pathway in yeast for the production of a safe, nonnarcotic antitussive and potential anticancer compound represents one such example.

**Figure 5:**
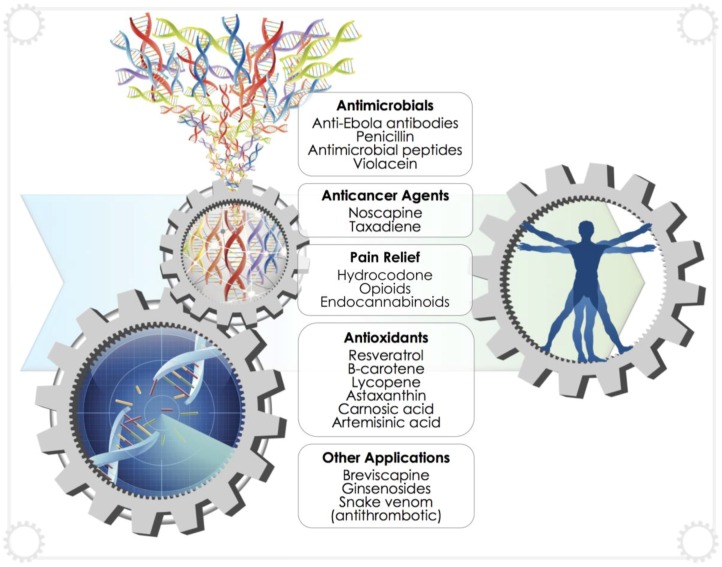
By harnessing the power encoded within genetic material, a wide-range of complex natural products may be produced by yeast cell factories for a diverse range of medical applications. Synthetic DNA is analogous to a computer code that directs the cogs or machinery of yeast cell factories to produce high-value products.

In recent years, we have witnessed several advances in the application of synthetic biology to produce biopharmaceuticals in yeast spurred by imaginative ideas and innovative approaches (Figure 4). The production of up to 25 g/L of artemisinic acid, a precursor for the antimalarial artemisinin, in *S. cerevisiae* demonstrates the power of synthetic biology approaches [9]. Since then, numerous researchers have combined synthetic biology approaches with more traditional metabolic engineering to produce a wide-range of medically-relevant compounds, including opioids, anti-cancer compounds, antimicrobials, antioxidants and even snake venom (Table 1; Figure 5). This section highlights some of these recent developments.

### 4.1. General Medical Applications

Opioids, obtained from the opium poppy (*Papaver somniferum*), are a class of compounds regularly used for pain relief. However, a global shortage of opioids in the developing world has led to efforts to identify cost-effective new sources of these molecules. Heterologous production of thebaine and hydrocodone was successfully undertaken in *S. cerevisiae* [47]. The strain housed a modular pathway assembly containing synthetic modules integrated into up to six different genome locations or on a yeast artificial chromosome (YAC). After constructing an initial yeast strain that produced thebaine, the pathway was further extended to produce hydrocodone [47]—notable for dispensing with the requirement to perform downstream chemical synthesis. While the biosynthetic production of these opioids would need to be improved by several orders of magnitude to be commercially viable [47], previous work undertaken on artemisinic acid [9], farnesene [48] and noscapine [8] suggest that it may be feasible to increase yield and optimise strains for commercial-scale production through further cellular engineering.

The plant *Erigeron breviscapus* is widely used in Chinese medicine and may be beneficial for the treatment of cardiovascular and cerebrovascular diseases [64,65,66]. However, supply of this plant is currently insufficient to meet demand [67]. The crude extract, breviscapine, primarily consists of the compound scutellarin and lower quantities of apigenin-7-O-glucuronide. Genomics analysis were applied to identify the complete metabolic pathway for biosynthesis of these molecules and applied synthetic biology approaches were used to produce 108 and 185 mg/L of scutellarin and apigenin-7-O-glucuronide, respectively, in *S. cerevisiae* [52]. Furthermore, an alternative to the use of plants for traditional Chinese medicine includes the biosynthesis of ginsenosides in yeast. These molecules, from the plant ginseng, may have antioxidant and anti-proliferative properties although their exact therapeutic benefit is yet to be elucidated [68]. Biosynthesis of the ginsenoside aglycons protopanaxadiol, protopanaxatriol and oleanolic acid in *S. cerevisiae* was undertaken using metabolic engineering approaches and the heterologous expression of several genes encoding plant enzymes [69].

The protease agkisacutacin is a component of pit viper (*Agkistrodon acutus*) venom and has potential as an antithrombotic drug [70]. Codon-optimised, gene-synthesised variants of the agkisacutacin α- and β-subunits were used to heterologously produce snake venom in *P. pastoris* [49]. Heterologous production of such compounds demonstrates the potential value of synthetic biology: it was calculated that 10 L of supernatant containing Agkisacutacin would replace the requirement to milk 15,000 snakes for 100,000 vials of snake venom [49].

Aside from illicit use, cannabinoids have been explored as therapeutic agents for the treatment of nausea and vomiting following chemotherapy, chronic pain or spasticity caused by multiple sclerosis [71]. Synthetic biology approaches for heterologous expression of genes encoding cannabinoids, with a focus on production in *S. cerevisiae*, were recently reviewed [72]. Biosynthesis of the Δ^9^-tetrahydrocannabinol precursor, Δ^9^-tetrahydrocannabinolic acid (THCA), was undertaken in *P. pastoris* [73], notably achieving production levels of 1 mM. Yields were later improved to achieve 3.05 g/L THCA in *P. pastoris* by overcoming bottlenecks through co-expression of genes encoding 12 helper proteins [60].

### 4.2. Anticancer Compounds

Cancer is a global disease that caused 8.8 million deaths in 2015, with incidences of cancer expected to grow by 70% over the next 20 years [74]. Although 60% of anti-cancer agents are derived from natural sources [75], their low titres have rendered their extraction from natural sources expensive and environmentally damaging. For example, the chemotherapy drug Paclitaxel (or Taxol) exists at a concentration of 0.02% dry weight in the bark of the Pacific Yew tree, necessitating the felling of several thousand trees to meet annual demand [76].

Although Taxol may now be retrieved from the needles of the European Yew, *Taxus baccata,* or through suspended cell culture [77], traditional methods for obtaining Taxol are insufficient to meet demand and so alternate sources of this compound are being sought [78]. A promising alternative to obtain Taxol is through microbial production. However, its heterologous production remains challenging in yeast, with only successful biosynthesis of the Taxol intermediate, taxadiene, reaching titres of 8.7 mg/L in *S. cerevisiae* [79]. These titres were later improved upon using synthetic biology approaches to reach 72.8 mg/L [59]. Nevertheless, these levels remain too low for commercial production from *S. cerevisiae* and further work is required to improve yield compared to the ~1 g/L titres obtained from engineered *E. coli* [80].

Potential novel anti-cancer agents include the phthalideisoquinoline alkaloid, noscapine, which is obtained from the opium poppy. Noscapine is commonly used as an antitussive, although evidence suggests that noscapine has more applications as an anticancer drug [81,82,83]. Noscapine has the advantages of being well-tolerated and displaying low-toxicity in patients. It was first shown to be produced in *S. cerevisiae* by using synthetic biology approaches to reconstitute a plant-based noscapine gene cluster from *P. somniferum* [84]. This work provided an experimental platform that elucidated the final three steps of the noscapine biosynthetic pathway, including the activity of an O-methyltransferase heterodimer. This platform also provided a means to synthesise other protoberberine compounds not found in nature, such as 1-hydroxycanadine. However, it should be noted that titres of noscapine produced by these yeast strains were low, and required the feeding of the expensive intermediate, canadine. More recent work by the same group demonstrated the complete de novo biosynthesis of noscapine from cheap carbon sources, and, through enzyme, pathway and strain optimisation, they observed an 18,000-fold improvement from initial titres to 2.2 mg/L [8]. This improvement notably includes a significant (300-fold) increase in noscapine production through the simple optimization of media and fermentation conditions, demonstrating that conventional attributes should not be overlooked during process optimisation.

### 4.3. Antivirals, Antibiotics and Antimicrobial Peptides

Recent work has demonstrated the production of antimicrobials and antivirals in yeast. An intriguing example is the production of a monoclonal antibody cocktail active against Ebola virus peptides in *P. pastoris* [55]. The 2013–2016 Ebola outbreak saw a rapid exhaustion in supplies of the ZMapp antibody cocktail, currently produced in the plant *Nicotiana benthamiana* [85]. A recombinase-mediated landing pad was used for genomic integration of genes encoding all three antibodies (2G4, 4G7 and 13C6) of the ZMapp cocktail [55], a potentially more efficient means of integration considering the low efficiency of homologous recombination in wild-type *P. pastoris*. The secreted proteins were then shown to bind directly to the Ebola virus, demonstrating that *P. pastoris* may be used as an alternative to produce antibodies. This synthetic biology platform was suggested to not only provide a reliable means for large-scale production of antibodies, but also to rapidly develop and screen novel forms to combat Ebola resistance [55].

Humanity faces a significant crisis from the threat of antibiotic resistance [86]. Synthetic biology is uniquely positioned to address this challenge, and will likely contribute to a rich source of new drugs in the year ahead (discussed in further detail in Section 5.1). One important class of antibiotics are the nonribosomal peptides. The first instance of producing the β-lactam antibiotic, penicillin, in *S. cerevisiae* using synthetic biology methods was recently described [36]. Penicillin productivity was increased by 50-fold using combinatorial pathway optimisation, with the resulting spent growth media displaying activity against *Streptococcus* bacteria.

Antimicrobial peptides (AMPs) provide broad-spectrum protection against parasites, fungi, bacteria and viruses and represent an alternative to the widespread use of antibiotics. However, they are extremely costly to chemically synthesise. *E. coli* has been used to produce AMPs, but their intrinsic lethality to their hosts necessitates their expression as fusion proteins [87]. A synthetic biology platform was recently developed for the production of the antimicrobial peptide, apidaecin Ia, in *P. pastoris* [88]. Apidaecin Ia is part of the apidaecins, originally identified in the honeybee, and displays the ability to inhibit growth of a range of Gram-negative bacteria [50]. Production volumes of > 700 mg/L and an 80% efficiency of bioactivity against *E. coli* were demonstrated [88].

### 4.4. Antioxidants

The stilbenoid class of compounds includes 3,5,4′-trihydroxy-trans-stilbene (resveratrol). Despite a lack of clinical trials in humans, experimental models do suggest that resveratrol may have some health benefits [89,90,91], although over-reaching media claims should be tempered with a degree of caution. Yeast strain engineering was used to achieve titres of 800 mg/L, the highest reported yield of resveratrol so far [57]. This work also included the introduction of novel heterologous enzymes for the de novo biosynthesis of the pinostilbene and pterostilbene resveratrol derivates. Production of resveratrol from yeast has been commercialised by Evolva Inc., (Reinach, Switzerland) (http://www.veriteresveratrol.com/), who apply synthetic biology approaches on a commercial basis.

The carotenoids are a class of compounds with health applications as antioxidants [92], but may also be beneficial for cardiovascular health or as anti-cancer agents. β-carotene is an antioxidant and a vitamin A precursor [93]. A combinatorial assembly-based approach using *Golden Gate* assembly was used to identify the optimal promoter-gene configuration for production of β-carotene in *Y. lipolytica* [37]. This approach demonstrated that an engineered lipid-overproducing strain, optimisation of growth conditions and multi-copy gene integration was required to produce the highest reported levels of up to 6.5 g/L and 89.6 mg/g of dry cell weight (DCW). Furthermore, the lycopene carotenoid has anti-cancer and antioxidant properties [94]. Through a combination of pathway engineering and host engineering, a lycopene yield of 55.56 mg/g DCW was achieved in *S. cerevisiae* [54]. Finally, a synthetic biology approach called Promoter-based Gene Assembly and Simultaneous Overexpression (PGASO) [95] was used to produce almost 1 mg/g DCW of the astaxanthin antioxidant in *K. marxianus* [51].

Carnosic acid is a diterpene obtained from sage and rosemary with a range of antioxidant applications [96] and may be heterologously produced in yeast using synthetic biology techniques such as *Golden Gate* assembly [97]. However, in some cases, a molecule of interest may not be the primary product of a pathway, and may be produced in low titres or in a mixture with other compounds. For example, synthetic biology techniques were applied to increase production of the pisiferic acid and salviol side-products in *S. cerevisiae* [53]. The carnosic acid pathway was engineered by introducing a single amino acid substitution in the key cytochrome enzyme, CYP76AH24, to shift the metabolic pathway away from carnosic acid and towards side-product formation. Salviol is potentially one of the active components of *Salvia miltiorrhiza* root extract [98], highly valued in traditional Chinese medicine, although its biological role is yet to be elucidated. Pisiferic acid has properties an antimicrobial agent [99].

## 5. Emerging Technologies and Future Trends for the Engineering of Synthetic Yeast Biofactories

Synthetic biology is a field in a rapid state of development, and so it is difficult to predict the state-of-the-art in the years ahead. However, several new technologies and trends have emerged in recent years which hint upon potential new applications for yeast synthetic biology and the production of high-value biopharmaceuticals. This section highlights recent developments in drug discovery, synthetic genomics and the home-brew of biopharmaceuticals at the point of care.

### 5.1. Yeast Synthetic Biology Expands the Biopharmaceutical Repertoire

An important attribute of evolution or in situ adaptation of plants to unfavourable environments [53] is to rapidly diversify the production of complex molecules. In their review, Breitling and Takano [100] described how synthetic biology may be applied to harness the inherent modularity of many natural biosynthetic pathways to generate new compounds. The authors also noted that synthetic biology can be used to mine the rich diversity of existing biosynthetic pathways in the natural world. Synthetic biology, especially when applied in yeast, holds great promise to expand chemical diversity and produce bioactive compounds with novel medical applications.

The biosynthesis of 74 compounds in *S. cerevisiae* were reported [101], of which > 75% had not been described previously. This synthetic biology platform applied a combinatorial genetics approach and an intracellular survival assay to produce a library of molecules with potential biological activity. The authors suggested that these compounds may form chemical scaffolds for future drug discovery through subsequent modification. Furthermore, a pressure test was recently undertaken by researchers at five separate institutions to make 10 molecules in 90 days in a range yeast and bacteria [102]. They were successful or close-to-successful for 6 out of the 10 molecules, and produced a vincristine (anticancer chemotherapeutic) intermediate in *S. cerevisiae*. In addition, the development of a heterologous expression (HEx) platform to rapidly identify and scale expression of genes encoding diverse fungal natural products in *S. cerevisiae* was recently reported [103]. Of the 41 biosynthetic gene clusters obtained from a wide range of fungal species, 22 were shown to produce a detectable product in *S. cerevisiae*. Overall, this approach presents a promising platform to identify novel bio-active compounds and awaken unexplored chemical diversity from cryptic biosynthetic gene clusters, which would not have been possible without the application of synthetic biology techniques including high-throughput DNA assembly.

Antibiotic resistance is one of the great challenges currently faced by humanity [86]. This is a challenge compounded due to a lack of new antibiotics: since the 1970s, only three new antibiotic classes have entered the market [104]. It is likely that synthetic biology will provide an important platform to develop new antibiotics in the years ahead. For example, the inherently modular nature of polyketide synthetase enzymes may be used in tandem with synthetic biology approaches to refactor polyketides for new activity [105]. Polyketide tailoring has already been applied in bacteria: pathway modularity was applied to generate erythromycin analogues in *E. coli* [106]. These molecules were modified through the activity of modular tailoring pathways and were shown to generate derivatives active against erythromycin-resistance *Bacillus subtilis*. A further example includes the biosynthesis of a glycopeptide antibiotic scaffold in the bacteria *Streptomyces coelicolor* [107]. By applying synthetic biology approaches, the authors introduced a series of scaffold-modifying enzymes in different combinations to generate a total of 15 molecules, nine of which had not been described previously and some of which displayed activity against glycopeptide antibiotic-resistant bacteria. 

Yeasts have the inherent advantage of resistance to antibiotics which may cause host lethality in prokaryotes. The biosynthesis of antibiotics in yeast is very much a nascent area of research, although a recent example includes the biosynthesis of penicillin in *S. cerevisiae* [36]. These authors suggested that yeast is an ideal testbed to accelerate the screening and diversification of biosynthetic pathways. Indeed, new nonribosomal peptide derivatives may be generated through the action of modular nonribosomal peptide synthetases and tailoring enzymes. The authors suggest that *S. cerevisiae* can be used to rapidly screen compounds for new activity and provide the means for true combinatorial synthesis of new nonribosomal peptides.

### 5.2. Synthetic Genomics, Sc2.0 and SCRaMbLE

**Figure 6:**
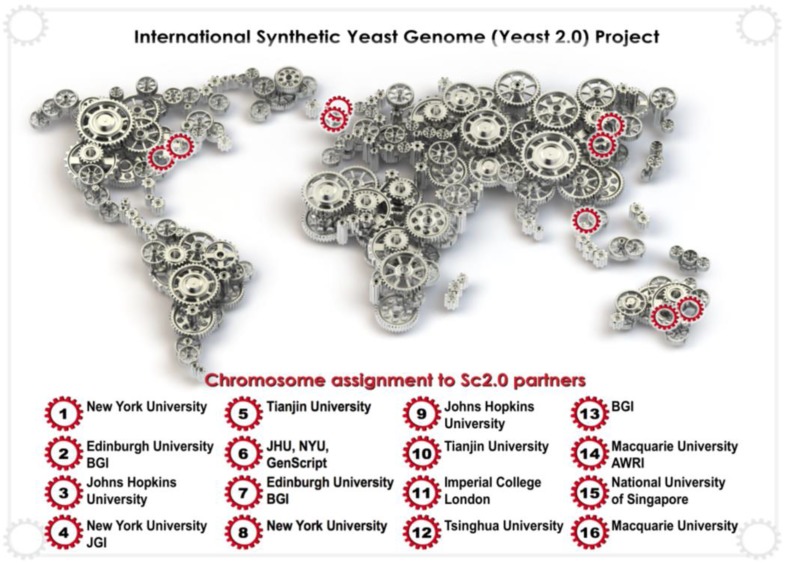
The Synthetic Yeast Genome (Yeast 2.0/Sc2.0) Project is an international endeavour. This coordinated project requires the concerted action of individual independently-operating gears to construct the sixteen chromosomes of the synthetic yeast genome. Each cog on the above figure represents a synthetic chromosome assigned to research groups affiliated with individual host institutions.

With recent developments in large-scale DNA synthesis, it is now possible to design and synthesise DNA on the scale of the genome. This has led to the ambitious Sc2.0 (or *Saccharomyces cerevisiae* version 2.0) project (Figure 6), which aims to design and construct a new version of the yeast genome [108]. Thus far, chemical synthesis of chromosome II [109], chromosome III [110], chromosome V [111], chromosome VI [112], chromosome X [113] and chromosome XII [114] are complete. This project demonstrates the power of successful collaboration between individually operating components or cogs of a larger apparatus.

A powerful attribute of this project is SCRaMbLE (Synthetic Chromosome Recombination and Modification by *LoxP*-mediated Evolution). *LoxPsym* sites are targets for the enzyme Cre recombinase, which, upon induction, may generate inversions, deletions, duplications and translocations in vivo. The introduction of *LoxPsym* sites throughout the synthetic yeast genome allow genome scrambling, theoretically leading to a near-infinite number of genome permutations. SCRaMbLE has been previously demonstrated to produce significant structural diversity in the right arm of synthetic chromosome IX (SynIXR) [115]. Further applications of the SCRaMbLE system were recently highlighted, including diploid and interspecies SCRaMbLEing [116], in vitro methods [117], a SCRaMbLE reporter system [118] and light-controlled recombination [119].

SCRaMbLE may also be applied to introduce new pathways for biopharmaceutical production. In conjunction with screening methods or a selection pressure, the surviving yeast cells will essentially choose the optimal genome configuration for pathway expression. This strategy was recently undertaken to increase production of penicillin and the antibiotic violacein from 2μ-plasmid-encoded pathway genes in a strain housing a SCRaMbLEd synthetic chromosome V (SynV) [56]. A SCRaMbLE-in method was also recently used to introduce genes encoding carotenoids and violacein into synthetic chromosomes [63] and iterative cycles of SCRaMbLE were used to increase carotenoid production ~39-fold (Multiplex SCRaMbLE Iterative Cycling (MuSIC); [120]).

**Figure 7:**
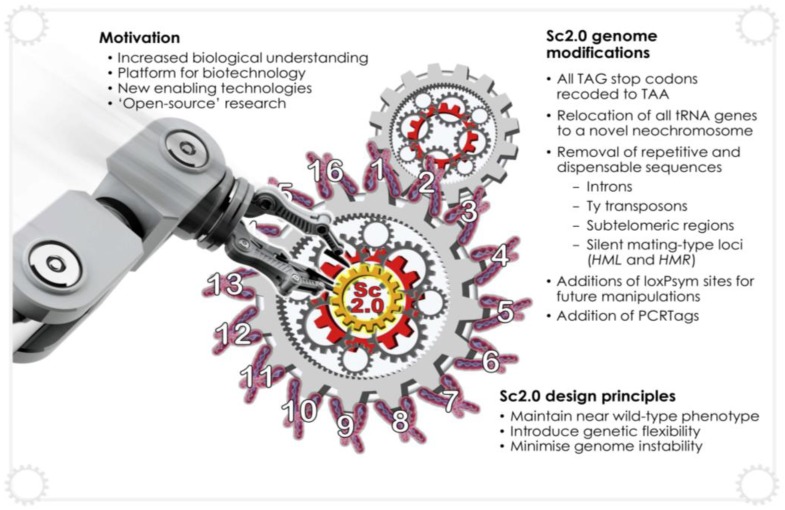
The Synthetic Yeast (Sc2.0) consortium is undertaking rapid progress towards the construction of a fully-synthetic eukaryotic genome. This project aims to provide new insight into the fundamental properties of eukaryotic biology through a build to understand approach facilitated by re-designing the underlying cellular machinery. To meet the central design principles, genome alterations include the introduction of LoxPsym sites for combinatorial rearrangement (SCRaMbLE), PCR tags for verification of synthetic DNA, stop codons recoded from TAG to TAA to allow future incorporation of artificial amino acids, the removal of retrotransposons, subtelomeric repeats and many introns and the relocation of tRNA genes onto a tRNA neochromosome.

A further possibility raised by the Sc2.0 project (Figure 7) is the construction of synthetic cells with bespoke function. In the future, it may be possible to rapidly construct synthetic yeast genomes that are designed for specific purposes, such as those optimised for the production of biopharmaceuticals.

### 5.3. Home-Brew Biopharmaceuticals

Synthetic biology presents the opportunity to address a global shortage of medicines [121], the production of which is generally expensive and requires large, complex infrastructure. In addition, many developing countries lack the facilities required to produce and store essential medicines. It would also be of significant benefit to rapidly produce vaccines at the point of outbreak, such those active against the Ebola virus [55], where the time-critical biosynthesis of vaccines at the source of demand would be of significant advantage. Recent developments in yeast synthetic biology suggest that home brew may be a potential approach to inexpensively produce biopharmaceuticals at the point of care. For example, a *P. pastoris* platform was developed to produce multiple biologics in a microbioreactor on the scale of a single dose [122]. This platform secreted human growth hormone and interferon alpha-2b in less than 24 h, with production of each controlled by a methanol-induced promoter and a novel β-estradiol-induced promoter. The same group [123] later evolved their method to describe the control over the ratio of biologics produced, consolidated bioprocessing to produce human serum albumin as a stabiliser for human growth hormone and single-batch biosynthesis and purification of two monoclonal antibodies.

Recent advances in producing opioids [47] and endocannabinoids [72] in yeast suggest that illicit home-brew drug production may become a reality in the years ahead. It has been argued that home-brew drugs should be regulated due to the risk of illicit use [124], although it should be noted that this technology still has some way to progress before illicit production, with no significant yields of opioids currently produced using engineered strains under home-brew fermentation conditions [125].

Synthetic biology will have numerous unexpected applications in the years ahead. An industrial brewing strain of *S. cerevisiae* was recently modified using synthetic biology techniques to produce monterpene aromatics mimicking hop aroma using synthetic DNA derived from yeast, mint and basil [126]. Additionally, a haploid *S. cerevisiae* wine strain was modified to produce a raspberry ketone above the flavour threshold in fermented wine must [127]. Perhaps, in the near future, one could functionalise beer or wine by brewing non-alcoholic variants to produce health-improving compounds in the supernatant? Assuming biopharmaceutical levels are above the therapeutic level, and that consumer perception becomes more amenable to the concept of genetically-modified beverages, a beer a day may well keep the doctor away.

## 6. Remaining Hurdles for Biopharmaceutical Production

Despite holding great promise to improve upon the production of biopharmaceuticals, yeast synthetic biology still must address similar challenges to that of traditional metabolic engineering. One of the most significant challenges yet overcome is the low yield/titre of biopharmaceutical compounds produced in yeast hosts, which ultimately limits the commercial viability of yeast production platforms. The factors affecting product yield/pathway efficiency and strategies to increase production have been reviewed previously [10,11,16].

General factors limiting production levels in yeast include metabolic burden caused by the redirection of host metabolic resources towards compound production, toxicity of pathway intermediates and products, secretion of pathway intermediates, precursor availability, cofactor imbalance, interference with host metabolic enzymes and inefficient enzyme activity in heterologous hosts [10,11,16]. Additionally, as metabolic pathways increase in size, their overall efficiency decreases proportionally [128].

Synthetic biology approaches (such as those described in Section 3) may be applied in conjunction with genetic engineering to address and potentially overcome several of these obstacles. These include the fine tuning/dynamic control over gene expression using synthetic promoters to improve flux, reduce metabolic burden and reduce intermediate toxicity [11,129]. Different combinations of promoters may also be introduced in a modular manner in tandem with combinatorial assembly to rapidly generate and screen optimal pathway configurations for improved compound production [36]. Semi-rational approaches, such as multiplex genome-scale engineering in yeast [39], also represent promising high-throughput approaches to improve the general traits of yeast production strains, such as stressor tolerance. Furthermore, the engineering of improved secretion in *S. cerevisiae* may be an avenue to improve compound yields [15]. Finally, the aforementioned compartmentalisation of metabolic pathways holds great promise to reduce intermediate toxicity and control local pathway environment.

Despite the comparatively recent advent of synthetic biology and the introduction of numerous new technologies and approaches, compound yields remain stubbornly low (Table 1). Given the inherent individual differences between each biopharmaceutical compound, it is unclear if there lies a singular magic bullet for improved yields, and so a combination of strategies may be required. However, synthetic biology is still a nascent field of research, and emerging technologies (detailed in Section 5), may yet lead to the development of robust and proven methodologies/workflows that apply a combination of approaches in a standardised manner. A greatly promising concept is that of the bio-foundry [130] or genome foundry. Rapid and inexpensive gene synthesis, in conjunction with automated DNA assembly and high-throughput screening methods, will facilitate rapid iterations through the Design-Build-Test cycle. Ultimately, the automation of synthetic biology approaches will lead to the predictive engineering of designer yeast strains with significantly increased capacity for biopharmaceutical production.

## 7. Synthetic Yeast Foundries Awaken Biological Potential

Yeast cells are the perfect biological machines capable of shifting the development and production of biopharmaceuticals into high gear. This minireview has highlighted the extensive diversity of biopharmaceuticals enabled by yeast synthetic biology and discussed new emerging technologies and trends in this rapidly-developing area. The natural world holds a diverse landscape of complex chemistry which may be harnessed for medical benefit. Yeast synthetic biology is ideally placed to not only awaken the cryptic diversity of complex natural products, but also to generate compounds with completely new biological activity. This nascent area of research is well positioned to address both existing and emerging human health challenges in the years ahead.

## Figures and Tables

**Table 1:** Examples of biopharmaceutical compounds produced by applying yeast synthetic biology.

Compound	Application	Compound Class	Chassis Organism	Titre	Natural Source
Agkisacutacin (Snake venom)	Antithrombotic	Protein	*Pichia pastoris*	100 mg/L [49]	*Agkistrodon acutus* (Pit viper)
Apidaecin Ia	Antimicrobial	Antimicrobial peptide	*P. pastoris*	>700 mg/L [50]	*Apis* (Honeybee)
Artemisinic acid	Artemisinin (anti-malaria) precursor	Sesquiterpene	*S. cerevisiae*	25 g/L [9]	*Artemisia annua* (Sweet wormwood)
Astaxanthin	Antioxidant	Carotenoid	*Kluyveromyces marxianus*	1 mg/g DCW [51]	Various, including krill and shrimp
Breviscapine (Scutellarin and apigenin-7-*O*-glucuronide)	Chinese medicine. Cardiovascular and cerebrovascular disease.	Flavanoid	*S. cerevisiae*	105 and 185 mg/L [52]	*Erigeron breviscapus*
Carnosic acid	Antioxidant	Diterpene	*S. cerevisiae*	18 mg/L [53]	*Rosmarinus officinalis* (Rosemary) and *Salvia officinalis* (Sage)
β-Carotene	Antioxidant	Carotenoid	*Yarrowia lipolytica*	6.5 g/L (90 mg/g) [37]	Various, including carrots
Hydrocodone	Pain relief (opioid)	Benzylisoquinoline alkaloids (BIA)	*S. cerevisiae*	<1 μg/L [47]	N/A (Semi-synthetic from Codeine)
Lycopene	Antioxidant, anti-cancer	Carotenoid	*S. cerevisiae*	55.56 mg/g DCW [54]	*Solanum lycopersicum* (Tomato)
Anti-Ebola monoclonal antibodies	Antiviral	Monoclonal antibody	*P. pastoris*	1 to 10 mg/L [55]	N/A
Noscapine	Anticancer	Benzylisoquinoline alkaloids (BIA)	*S. cerevisiae*	2.2 mg/L [8]	*Papaver somniferum* (Poppy plant)
Penicillin	Antibiotic	Beta-lactam nonribosomal peptide	*S. cerevisiae*	14.9 ng/mL [56]	Penicillium fungi
Pisiferic acid	Antimicrobial agent	Diterpene	*S. cerevisiae*	2.65 mg/L [53]	*Chamaecyparis Pisifera* (Sawara cypress)
Resveratrol	Several; antioxidant	Stilbenoid	*S. cerevisiae*	800 mg/L [57]	*Polygonum cupidatum* (Japanese knotweed)
Salviol	Established bioactivity, awaiting further evaluation	Diterpene	*S. cerevisiae*	15 mg/L [53]	*Salvia miltiorrhiza* (Chinese sage)
Strictosidine	Intermediate	Monoterpene indole alkaloid	*S. cerevisiae*	0.8 mg/L [58]	N/A (chemical synthesis)
Taxadiene	Anticancer Taxol precursor	Diterpenoid	*S. cerevisiae*	72.8 mg/L [59]	*Taxus brevifolia* (Pacific yew)
Δ^9^-tetrahydrocannabinolic acid	Tetrahydrocannabinol precursor	Cannabinoid	*P. pastoris*	3.05 g/L [60]	*Cannabis sativa* (Cannabis)
Thebaine	Opioid precursor	Benzylisoquinoline alkaloids (BIA)	*S. cerevisiae*	<1 μg/L [47]	*Papaver somniferum* (Poppy straw)
Theophylline	Anti-asthma medication	Methylxanthine	*S. cerevisiae*	61 μg/L [61]	*Camellia sinensis* (Tea) and *Theobroma cacao* (Cocoa)
Vindoline	Anticancer (vinblastine and vincristine) precursor	Monterpenoid indole alkaloid	*S. cerevisiae*	2.7 mg/L [62]	*Catharanthus roseus* (Madagascar periwinkle)
Violacein	Antibiotic	Bis-indole pigment	*S. cerevisiae*	16.8 mg/L [63]	*Chromobacterium violaceum*
